# Feedback on the FDA's February 2006 draft guidance on Patient Reported Outcome (PRO) measures from a developer of PRO measures

**DOI:** 10.1186/1477-7525-4-78

**Published:** 2006-10-09

**Authors:** Clare Bradley

**Affiliations:** 1 Health Psychology Research, Royal Holloway, University of London, Egham, Surrey TW20 0EX, UK

## Abstract

I believe that the FDA guidelines have already had an impact in encouraging good practice in the use of PROs. There are, however, important improvements that need to be made to the guidelines, particularly in the use of health status and quality of life terminology. It is essential to distinguish between health status and quality of life and to use both terms. Nothing is to be gained and a great deal will be lost if the term quality of life (which has been misused as an umbrella term in the past) is abandoned and replaced with the term health status. Patients want us to consider their quality of life as well as their health. To abandon the term would be to forget about their quality of life and focus only on their health. Patients are well able to tell us what quality of life means to them and to rate the impact of a condition on their quality of life if we use individualised quality of life measures and individualised condition-specific quality of life measures to allow them to do so. Although my experience with PRO measures would support many of the recommendations in the guidelines there are others that I would not fully agree with or would contradict on the basis of my own research evidence. I have provided references to that research and hope that the FDA will feel able to do the same when they finalise their guidelines.

## Introduction

The US Department of Health and Human Services Food and Drug Administration (FDA) made public in February 2006 a document entitled 'Guidance for Industry Patient-Reported Outcome Measures: Use in Medical Product Development to Support Labeling Claims DRAFT GUIDANCE'. I have a particular interest in this draft guidance as I specialise in the design, development and use of PRO measures and license them for use in clinical trials, other research and routine clinical practice. My measures include:

• the Diabetes Treatment Satisfaction Questionnaire (DTSQ) in its status (DTSQs) and change (DTSQc) forms [1-6] and related measures for other conditions including the HIVTSQ, RTSQ, RetTSQ, GHerpTSQ, ThyTSQ [7-12], and the newly designed DTSQ-Teen and DTSQ-Parent. The DTSQs and DTSQc are fully linguistically validated in 65 language versions

• the Well-being Questionnaire (e.g. W-BQ12) [3,13-18] generic measure of well-being is psychometrically validated for a range of populations including those who have diabetes (type 1 and type 2) macular disease and growth hormone deficiency and fully linguistically validated in 37 language versions

• the ADDQoL measure of the impact of diabetes on quality of life [4,19] with related measures for other conditions including RDQoL, RetDQoL, MacDQoL, HDQoL, A-RHDQoL, ThyDQoL, ADDQoL Teen [12,20-27] and recently designed ADDQoL Jnr (for 5–8 year olds) and ADDQoL Jnr+ (for 9–12 year olds). The ADDQoL, MacDQoL and RetDQoL are linguistically validated in 16–25 language versions.

I welcome the FDA guidance as a much needed source of information about the standards required in PRO design, linguistic validation and psychometric validation. I recognise that the guidance may be very useful in encouraging good practice.

I was one of the 56 individuals/organisations who submitted comments on the draft FDA guidelines by their deadline of 4^th ^April. My original comments can be viewed with others on the FDA website [28]. I also attended a meeting in Washington DC on 29^th ^June 2006 organised by ISOQOL (the International Society for Quality of Life Research) where the FDA was represented. I had expected this meeting to facilitate discussion of the draft guidelines with the FDA.

Unfortunately the FDA representatives stayed only long enough to give their own opening presentations and respond to specific questions arising from those presentations but left before the following talks by ISOQOL members with lifetimes of experience of working with PROs providing feedback on the draft guidelines and suggestions for improvements. The FDA missed out on a valuable opportunity to learn from experts in the field. There is to be a follow up meeting before the ISOQOL conference in Lisbon in October and it is to be hoped that the FDA will come to listen as well as to speak. It was heartening to find that the FDA did appear to have taken note of some of the feedback previously provided and they encouraged submission of further written comments even though the original deadline is passed. Here I select some of the key issues raised in the comments I submitted to the FDA and provide some input following the Washington meeting.

## Discussion

### References

First it should be noted that the FDA provided no references in their guidelines. It is unclear why they feel it is unnecessary to back up their statements with evidence and examples. Their guidelines will be a great deal more credible and helpful in improving standards applied in developing and using PROS when properly referenced.

### Terminology, in particular 'health status' and 'quality of life'

I welcome the use of the term '*patient-reported outcome (PRO) measures*' in place of the ubiquitous and, usually inaccurate, use of '*quality of life measures*'. Previously '*quality of life*' was used as an umbrella term to cover a wide range of PRO measures including health status instruments which are actually measuring quality of health and not quality of life. This distinction is important and failure to recognise the difference has led to some highly misleading conclusions and misguided policies. I emphasised the importance of distinguishing between quality of life and health status in a commentary in the Lancet in 2001 [29] where I gave the example of the influential UK Prospective Diabetes Study (UKPDS) as one which used health status measures but in the published report the authors interpreted their findings as if they were measuring quality of life [30]. The UKPDS authors concluded that intensified treatment for Type 2 diabetes had no impact on quality of life and recommended widespread use of intensified treatment. In fact their findings showed there was no impact of intensified treatment on patients' perceptions of the quality of their health and their quality of life was not measured. This is a very different conclusion and a far less desirable one than the one reached erroneously by the UKPDS authors.

Line 31 of the draft guidance defines a PRO as '*a measurement of any aspect of a patient's health status that comes directly from the patient...*'. It seems that the FDA was here misusing the term '*health status*', in the way that previously the term quality of life was misused, as an umbrella term to encompass a variety of other outcomes, which lines 35 and 36 suggest include symptoms, activities of daily living and quality of life. Thus quality of life measures are here conceptualised as a subset of health status measures instead of health status being (wrongly) seen as one of a range of quality of life measures and still there is no recognition of the importance of distinguishing between these two key concepts, health status and quality of life.

If the term '*health status*' is upgraded in this way to take over from '*quality of life*' as an umbrella term we will have as much, if not more, confusion over terminology and, worse still, patient reported outcomes will come to be seen as measures of health as viewed by the patient. There is a danger that clinical trials will be satisfied with measuring patients' reports of symptoms and will fight shy of measuring what the FDA are describing as '*extremely complex concepts such as quality of life*'. The great advantage of the term '*PRO*' is that it is a neutral term that covers all patient reported outcomes including their satisfaction with their treatment, their well-being, their quality of life and their symptoms and health without needing an interim term such as '*health status*' to limit the definition. If the FDA really feels the need to describe the kind of outcomes that PROs refer to then they might consider '*health and quality of life outcomes*' which is the phrase eventually agreed upon as the title of the present journal. This phrase makes it clear that health outcomes are one form of PRO and quality of life outcomes are another and both are important but different, an issue discussed in the first editorial of the journal written by myself and the editor, Marcello Tamburini [31].

However, in many ways it is more comprehensive and simpler to refer just to '*Patient Reported Outcomes*' and make it clear that PRO measures can include measures of symptoms, health status, treatment satisfaction, well-being and quality of life. At the Washington meeting, the three representatives from the FDA, Sahar M Dawisha, Edwin P Rock and John Powers, gave three interrelated presentations on the thinking behind the guidelines in preparation. It was encouraging to see that they were no longer defining a PRO as '*a measurement of any aspect of a patient's health status*': perhaps they were making good use of our written comments. Instead, PRO was defined as an *'element of feeling or function affected by disease, reported directly by patients'*. While welcoming this broader definition that does not focus entirely on health status it is still unnecessarily limited in focusing on '*feeling or function*' (what about cognitions and knowledge/understanding of the treatment and condition, adherence and self-care behaviours?) and only those that are affected by disease (what about the effects of treatment?). I suggest that PROs are well-enough defined by their name – outcomes reported by patients – if the FDA feels the need to narrow down the kind of PRO measures that they accept they can do so without interfering with the definition of a PRO.

### Conceptualising quality of life

The guidance described the concept of quality of life as *'extremely complex'*. The FDA offered the following unhelpful definition of Quality of Life in the glossary: *'A general concept that implies an evaluation of the impact of all aspects of life on general well-being. Because this term implies the evaluation of nonhealth-related aspects of life, it is too broad to be considered appropriate for a medical product claim'*. Surely it should be the ultimate aim of a treatment to benefit patients' quality of life? We may sometimes have to settle for reducing the damage done to quality of life by a medical condition such as diabetes but we will still need a definition of quality of life. If we try to define what quality of life is in a way that is appropriate for everyone it is indeed a very complex and perhaps an impossible task. However, if we follow the advice of Dick Joyce and define quality of life in terms of what the individual thinks it is [32] and measure it using individualised measures (e.g. [19,33]), it becomes a manageable, measurable and useful concept. It is very important for patients that clinical trialists do not duck the issue of measuring the impact of new treatments on the quality of life of individual participants in trials and measure only the quality of their health. It is the bigger issue of quality of life that is most important to patients and it makes a great deal of difference to them if new treatments impair their quality of life or improve their quality of life. Only the patients can tell us how a treatment affects their quality of life using individualised PRO measures designed for the purpose (e.g.[19-22,25]).

My ADDQoL questionnaire provides an overview question about quality of life per se as well as measuring the impact of diabetes on quality of life. There have been several studies which have shown significant benefits to quality of life on the overview present quality of life item as well as reductions in the negative impact of diabetes on quality of life (e.g. [34,35]). The definition of quality of life that I used as a basis for the design of the ADDQoL and related measures is that recommended by Joyce: quality of life is what the individual thinks it is. The individual's view of their quality of life may indeed include aspects of life that are not health related although as medical conditions become more severe and/or their treatment becomes more demanding and/or invasive, the aspects of life that are not health related diminish.

I believe it is essential that we face up to the importance of defining and measuring quality of life *per se *and do not avoid the issue or confuse matters further, as the FDA did, by defining quality of life in terms of well-being but then not defining well-being in their glossary! There are many measures of well-being which typically include subscales to measure depression and anxiety, energy, and, sometimes, positive well-being (e.g.[13,36]). When a person is depressed and anxious their quality of life is also likely to be impaired. However, someone who is not depressed or anxious may nevertheless feel that their quality of life would be much improved if they didn't have diabetes. Thus measures of well-being are no substitute for measures of quality of life. I recommend that the FDA adopt a simple patient-centred definition for the concept of quality of life – quality of life is what the individual concerned thinks it is [32] and encourages the considerable efforts made to date to measure individualised quality of life (e.g. the SEIQoL [33] which is the focus of a special interest group in ISOQOL) and the impact of medical conditions on individual's quality of life (e.g. the ADDQoL [19] which has already been welcomed by several reviewers despite the first publication of the ADDQoL being only six years old [37-39]).

### Health-related quality of life (HRQL)

HRQL was defined in the draft guidelines as '*A multidomain concept that represents the patient's overall perception of the impact of an illness and its treatment. An HRQL measure captures, at a minimum, physical, psychological (including emotional and cognitive), and social functioning. Claiming a statistical and meaningful improvement in HRQL implies: (1) that the instrument measures all HRQL domains that are important to interpreting change in how the study population feels or functions as a result of treatment; and (2) that improvement was demonstrated in all of the important domains.*'. This definition would seem to allow for some health status measures to be classed as HRQL measures (e.g. SF-36) for some patient groups as well as condition-specific quality of life measures (e.g. ADDQoL [4,19]). This is because the FDA refers to the patients' perception of the impact of an illness and its treatment but does not make explicit whether that impact is on their health or on their QoL. I think this encourages health status measures to be mislabelled as if they were quality of life measures (or health-related quality of life measures) when they are more accurately construed as measures of the quality of health and creates problems of interpretation discussed above and elsewhere [29].

Point (1) above will exclude many generic tools which do not adequately assess the impact of specific conditions on aspects of life important for quality of life: this may be an important step forward. For example, the aspect of life measured by the ADDQoL that is most impaired by diabetes is *freedom to eat as I wish*, is not measured by any other quality of life measure that I know of. Awareness of this major influence of dietary restrictions on quality of life led to the evaluation of the DAFNE (Dose Adjustment For Normal Eating) approach to insulin treatment for diabetes with major benefits to quality of life, treatment satisfaction, well-being and glycaemic control [35]. The DAFNE approach was supported by the recent National Service Framework for Diabetes in the UK and the Department of Health funded roll out of the approach nationwide. The value of DAFNE would not have been demonstrated by generic health status tools such as the EQ5D or the SF-36 which are all too often mislabelled HRQoL measures.

Point (2) above: I would take issue with the suggestion that improvement needs to be demonstrated in all of the important domains in a HRQL instrument. First because improvement can only be demonstrated in domains where deficits are apparent to start with, however important the domain may be, and we cannot expect that deficits will always be found for all important domains in all uses of a questionnaire. Secondly it seems unreasonable to expect to see benefits for all important domains even if there were deficits to start with. The outstandingly successful DAFNE approach did not achieve significant improvements for all the domains of the ADDQoL even though it showed significant benefits on the overall score and on many specific domains [35]. Just as we wouldn't reject a diabetes treatment because lipids did not improve alongside improvements in blood glucose control so too we should not reject a treatment because not all domains of a PRO measure improve.

### Quality of life in the taxonomy of PROs

Quality of life does not appear in Table 1 of the draft guidelines. The use of the term '*Overall health status*' rather confirms my concern that the FDA was substituting global misuse of the term '*quality of life*' with global misuse of the term '*health status*'. Health status measures can be useful but they are not everything. Quality of life and health-related quality of life measures are an essential subset of PRO measures for which health status measures provide no substitute. It is to be hoped that the FDA will continue to revise their definitions and taxonomy and, like ISOQOL, will recognise the value of individualised quality of life measures.

### Modification of PRO instruments

I welcome efforts to discourage users of established validated instruments from tinkering with the wording of questionnaires unnecessarily while referring to the validation of the original instruments as evidence for the modified instrument's validity and reliability (lines 176–181). However, with some instruments, such as the DTSQc, it is necessary to modify the instructions to relate specifically to the conditions of the clinical trial in which it is being used and we now have considerable evidence to show that the psychometric properties of the DTSQc remain robust to such changes [5,6]. I encourage users of the DTSQc to check the psychometric properties on each new use but would not go as far as to say that each new use (with modified instructions) should be treated as if it is a new measure.

### Comparison of present state with an earlier state

In lines 339 to 343 the FDA warned against instruments that rely on patients' memory in recalling experiences over a period of time: '*It is usually better to construct items that ask patients to describe their current state than to ask them to compare their current state with an earlier period..*'. While this may sometimes be good advice there are exceptions. Where measurement of patient satisfaction with treatment is concerned we often find that patients report being very satisfied with their current treatment until they experience a better treatment and then they want to be able to say that they are much more satisfied with the new treatment. If they have been given a status measure of treatment satisfaction at baseline and have given optimum responses showing they are very satisfied, they will not be able to respond any more positively at follow up when they are using a new treatment with which they are much more satisfied. It was to overcome such ceiling effects with the DTSQs status measure of satisfaction with diabetes treatment that I designed and developed the DTSQc measure of change in treatment satisfaction for use at follow up. This allows patients to say that they were very satisfied at baseline with the treatment they were using prior to the trial but are very much more satisfied with the new treatment they experienced within the trial. The DTSQc is also useful in crossover trials [6]. We are finding that the DTSQc provides valuable data when used in addition to the DTSQs and overcomes ceiling effects that are sometimes found when the DTSQs is used alone [5,6].

### Asymmetric response options

Lines 367–369. It is suggested quite appropriately that response options should not bias the direction of responses. However, the example given suggests that offering one negative choice, one neutral choice and two or more positive choices on a scale will make it more likely that patients will respond that they feel or function better. We have actually changed the symmetric response options originally used in the MacDQoL to asymmetric response options because respondents rarely used the response options which indicated that their quality of life would be worse if they didn't have macular disease as few people see any benefits of having this degenerative eye condition [22,23]. It is true that patients are more likely to respond in the direction that has more response options but this was the reason for making the scale asymmetric and not the result of asymmetry.

### Weighting

Lines 416 to 419. *'Equally weighted scores for each item are appropriate only when the responses to the items are relatively uncorrelated. Otherwise the assignment of equal weights will overweight correlated items and underweight independent items.' *William Lenderking and colleagues in their Washington presentation asked '*Does this statement imply that uncorrelated items should be grouped together (hence undermining internal consistency), or simply that redundant domains should not be included?*' and recommended that the FDA's draft passage on weighting be deleted. I agree. If the FDA wish to evaluate the role of weightings further then they might consider individualised condition-specific quality of life measures where the rating of impact of the condition on each life domain is weighted by the individual's rating of the importance of the domain to the individual's quality of life. Such weightings have been shown to alter substantially the rankings of weighted impact scores across domains compared to unweighted impact scores [19,40]. Weightings here play a key role in conveying the individual's view of the impact of their condition on their quality of life.

### Minimum important difference (MID)

Table 4. I agree that it can be helpful to consider the MID for clinical measures which are intermediate outcomes that may not be important outcomes for the patient in themselves but only in so far as they are predictors of other outcomes that are important (e.g. HbA1c measures of blood glucose in diabetes). With some PRO measures that ask about symptoms, health status or visual functioning without asking about the importance of the issue in question for the patient, it may also be useful to determine MID. However, a statistically significant difference on measures of treatment satisfaction that have been designed explicitly to measure issues of importance to patients (e.g. DTSQ) will necessarily be an important difference. So too will be a statistically significant difference on an individualised measure of the impact of a condition on quality of life, where the importance of an aspect of life for an individual's quality of life is part of the assessment (e.g. ADDQoL).

I was not impressed with the list of ways in which people have attempted to derive MIDs that the FDA has reviewed and the comments made by the FDA suggested that they have serious reservations too (lines 554–564). I also have major concerns about the first method outlined (551–554) which was not commented on by the FDA. This method involved mapping changes in PRO scores to clinically relevant and important changes in non-PRO measures and suggests that PRO measures be judged by their similarity to non-PRO measures such as spirometry scores in asthma. While it may be appropriate to expect some PRO measures such as those measuring health status or visual function to map onto clinical measurements, it is not appropriate for other PROs such as patient satisfaction or well being or the impact of the condition on quality of life which depend on much more than the clinical outcomes achieved. These latter PROs will depend on the demands of treatment and the extent to which the treatment can be adapted to suit the individual without damage to quality of life. It is crucial that we should be able to measure these PROs without being required to show that they map onto non-PROs! Indeed, it is perfectly possible that despite bringing about improvements in clinical outcomes a new treatment causes greater negative impact on treatment satisfaction and quality of life and, if so, patients are unlikely to be able to maintain clinical improvements in the long term.

### Linguistic validation (LV) of PRO measures

Some pharmaceutical companies who have previously paid only lip service to the need for linguistic validation, are now accepting that this is a task for specialists who will take 5 months to conduct a full linguistic validation of a questionnaire into another language. I have long collaborated with Mapi in Lyon on LV work. There are cheaper competitors who will complete the work in a fraction of the time, but experience has shown me that this is a false economy as high standards cannot be met at such speed. I think it would be helpful to provide rather more guidance on the quality of LV work required to produce good translations of PRO measures. In particular it would be helpful to note that it is good practice for the developer of the measure to be closely involved in the LV work. I employ a full-time linguist to manage my collection of translations and she and I are actively involved in LVs of my questionnaires. Even so, I still recommend that confirmatory factor analysis be used to check the psychometric properties of the new translations when first used.

### Blinding and randomisation

I must take issue with the statement that '*open-label studies, where patients and investigators are aware of assigned therapy, are rarely credible*' (line 717–8). In chronic disorders such as diabetes, all participants in trials will receive active treatment and the issue is more often whether they receive a new treatment or continue with an existing treatment rather than whether they receive active treatment or placebo. New treatments may carry risks and possible unwanted effects as well as benefits and it is not appropriate to assume that patients will always be more positive about a new treatment than about an old treatment. It is said on line 721 that '*Every effort should be made to assure that patients are masked to treatment assignment throughout the tria*l'. In practice this may mean that patients are asked to use two treatments, one of which is a placebo. This places additional demands on the patients that do not reflect the clinical realities of either treatment and render the trial unsuitable for evaluating the impact of treatments on patient satisfaction or quality of life. While I agree that '*The impact of unblinding is important to consider in the interpretation of study results*' (line 723) it is equally important to consider the impact of blinding on study results. Blinding should not be assumed to be universally desirable and in itself can distort study results.

John Powers from the FDA in his introductory talk in Washington referred to a paper by Iain Chalmers and colleagues [41] which compared trials that were more rigorously blinded with those that were less so and showed that the less rigorously blinded or open trials reported bigger effect sizes. The assumption made by Iain Chalmers and colleagues and by John Powers seemed to be that unblinded trials overestimate treatment effects compared with blinded trials but no support was offered for this assumption. It is equally possible that artificially blinded trials, that abandon external validity in their efforts for control, underestimate treatment effects compared with more naturalistic unblinded trials which provide more valid estimates of treatment effects.

Line 726 suggested that '*questions that ask how patients' current status compares to baseline seem likely to be more influenced by unblinding (optimism can readily be expressed as a favourable comparison) than questions about current status (which requires a current assessment, not a statement about duration)*' (I think the FDA probably mean '*differences*' rather than '*duration*' here.) It is particularly frustrating that there is no reference given for evidence for this point. In my experience of using the DTSQs (status measure) and the DTSQc (change measure) we often see that the DTSQc shows greater improvements in satisfaction with treatment than are shown by the DTSQs. However, separate analysis of patients who scored at or near ceiling on the DTSQs at baseline and patients who had more room to show improvement in satisfaction showed clearly that ceiling effects were limiting the benefits shown when the status measure alone was used and the DTSQc provided a more accurate representation of the benefits patients experienced [5]. It is possible that other studies showing fewer benefits with status measures than with change measures are in fact underestimating the benefits of treatment due to ceiling effects with the status measures that are overcome by using change measures.

### Statistical considerations for patient-level missing data

Line 1004 refers to imperfect strategies that 'try to predict missing outcomes for a patient who has withdrawn from the trial using data from subjects* who stayed in the trial and for whom all data have been collected'. Participants who withdraw from trials are likely to have worse scores on PROs such as treatment satisfaction measures than are those who continue in a trial and to impute missing values for those who withdraw from those who remain is likely to overestimate patient satisfaction. It would be much more informative to give the PRO measure to participants who withdraw early or to include interim data collections of PROs for use in endpoint analyses.

### * Subjects

The British Psychological Society advises that the term '*subjects*' not be used as it can cause offence and suggests to some potential participants that they may be subjected to unpleasant experiences. The FDA might wish to refer to '*participants*', '*respondents*' or '*individuals*' instead.

### Copyright

No mention is given of the need to respect copyright in questionnaires. There has recently been an increased demand for linguistic validation certificates documenting the procedures used in the linguistic validation work to produce new language versions of questionnaires and I believe this has been driven by the demands of regulatory bodies. It certainly seems to be a useful way of discouraging unauthorised translations and ensuring that only authorised translations are sought and used. The FDA is in an excellent position to encourage good practice in obtaining PROs from copyright holders and discouraging unlicensed use.

## Conclusion

PRO is a useful, broad term and it is not helpful to limit the definition either by suggesting all PROs are measures of health status (as the FDA did in the draft guidance) or by defining PROs as '*feelings or function*' affected by disease (as they did at the Washington meeting). It is essential that quality of life and health status are seen as two different kinds of PRO and that neither be used to subsume the other. If health status is used as an umbrella term it will wrongly suggest that QoL is determined exclusively by health and will do nothing to discourage the all-too-widespread practice of measuring quality of health and misinterpreting the findings as if they were measuring QoL. The FDA added confusion rather than clarity with their glossary definition of QoL in terms of well-being which remained undefined. If we define QoL as 'What the individual thinks it is [32] and measure it using individualised measures (e.g. [19,33]) it is a useful concept and arguably the most important outcome measure of all. The FDA's definition of the term HRQOL obscured the essential difference between health status measures and QoL measures by omitting to specify that the perceived impact to be measured should be the impact of the condition and its treatment on QoL (HRQoL) rather than simply the impact of the condition and its treatment on health (health status). It may be necessary to abandon the much abused term HRQoL for a while in favour of '*condition-dependent QoL*' or '*condition-specific QoL*' to break the widespread habit of wrongly referring to health status tools as HRQoL measures.

Key aspects of the draft guidelines discussed here included the following:

• I have taken issue with the FDA's suggestion that improvement needs to be demonstrated in all of the important domains of a '*HRQoL*' instrument. I can see no basis for such an all-or-nothing approach for either health status or QoL instruments, and no precedent in requirements for clinical measures.

• The FDA warned against instruments that ask patients to make direct comparison of their current state with a previous state. I have argued that change measures are valuable in overcoming ceiling effects common in status measures of treatment satisfaction and can provide more, not less, accurate estimates of change experienced [5,6].

• I disagree with the FDA's view that asymmetric response options necessarily bias responding and give evidence for the value of such asymmetry. I also provide evidence for the value of weighting in conveying individuals' views of the impact of their condition on QoL.

• Efforts to measure minimally important differences recounted by the FDA were not impressive and I emphasise that it is crucial that we should be able to measure PROs without being expected to show that they map onto biomedical measures.

• I welcome the discussion of the importance of linguistic validation (LV) of PRO measures for use in multi-national trials though suggest that there needs to be more guidance on the quality of LV work which ideally should involve the developer and include patient testing if optimal quality is to be achieved. The requirement of some regulatory bodies for LV certificates from the copyright owner which document the LV work conducted to produce each translation is particularly useful in raising awareness of the LV work needed and in preventing unauthorised translation and use of questionnaires – a problem that the FDA might helpfully discourage.

• Dismissing open-label trials as '*rarely credible*', the FDA revealed an uncritical view of '*blinding*' as a universal benefit when blinding can cause more problems than it solves when PROs are a focus of trials. In evaluating the impact of treatment on treatment satisfaction and other PROs it is essential that the treatment reflects clinical realities without the addition of placebo treatments in attempts to mask treatment assignment.

• Missing data are also problematic for interpreting PROs but substituting data from those who complete the trial will mislead as their outcomes are likely to be more positive than those who discontinue. Better to give PRO measures to participants who withdraw early or bring forward interim data for use in endpoint analyses.

• Finally, it is essential that the FDA provide references to support the advice they give in their guidance if it is to be useful in improving the standards applied in developing and using PROs.
